# Editorial: Multi-Modal Imaging in Neurological Conditions: Translational Applications

**DOI:** 10.3389/fneur.2022.855122

**Published:** 2022-02-15

**Authors:** Maria Petracca

**Affiliations:** Department of Human Neurosciences, Sapienza University of Rome, Rome, Italy

**Keywords:** multi-modal imaging, quantitative MRI, functional MRI, spectroscopy, fixel-based analysis, glutathione imaging

Multi-modal imaging represents the ideal approach for the investigation of complex brain disorders. Thanks to the application of diverse imaging techniques and parameters, mechanisms sustaining disease development and progression can be evaluated from different points of view, gathering information on several aspects of brain structure and function (1). This approach offers the invaluable possibility to simultaneously assess abnormalities related to different pathophysiological pathways, characterize their interaction, and capture clinically meaningful features. Allowing for the rapid collection of a large amount of high-quality quantitative data, that can be used for the selection of relevant information, multi-modal imaging is to neuroscience what drone technology is to archeological site mapping. This Research Topic, hosting 3 review articles and 5 original articles, offers comprehensive overviews of multi-modal imaging applications, as well as experimental examples illustrating how multi-modal imaging can help us to (i) gain insight into the physiopathology of brain disorders and clinical deficits and (ii) identify biomarkers that might guide patient stratification for prognostic and therapeutic purposes.

The review article by Seiler et al. summarizes quantitative MRI findings in inflammatory, cerebrovascular and neurodegenerative disorders, highlighting the presence of similar pathological substrates across different, unrelated morbid conditions. Indeed, the determination of quantitative tissue parameters enables the detection of early abnormalities and the characterization of microstructural correlates of clinical manifestations. However, although of undisputable interest, the characterization of brain microstructure should always be interpreted after careful consideration of the intrinsic limitation of each method (2) and in light of the impact exerted by concomitant pathological processes, as, for example, in the case of atrophy and iron content/concentration (3).

The second review, by Sen et al. describes the pivotal role of multi-modality imaging in urea cycle disorders, focusing on MR spectroscopy and functional near infrared spectroscopy, which, assessing local changes in cerebral hemodynamic levels of cortical regions, represents a valuable surrogate to functional MRI (fMRI). The combination of spectroscopy and fMRI is particularly intriguing, as it allows to explore the relationship between neurotransmitter levels and cortical function (4). Finally, Brown et al. focus on the clinical value and pathological meaning of multi-modal imaging, assessing improvements gained by utilizing a multi-modal approach in lieu of a single modality in Down's syndrome. Not only the Authors highlight the insights that might be gained through each method and their combination, but also discuss the relevance of ultra-high field MRI, whose application in clinical research has been a game changer for neuroimaging applications in epilepsy, brain tumors, dementia, neuro-psychiatric disorders, and multiple sclerosis (5). Indeed, the association of advanced imaging methods with ultra-high field MRI offers an invaluable tool to investigate disease pathophysiology, monitor disease progression, and treatment response (6).

As per the investigation of structural modification behind clinical deficits, Finkelstein et al. explored axonal degeneration in HIV patients at risk for neurocognitive impairment *via* fixel-based analysis, a recent diffusion-based technique that models individual fibers at the sub-voxel level. In this report, microstructural abnormalities in different white matter bundles are able to distinguish between cognitively normal patients and those with cognitive impairment, in line with the recently confirmed central role of white matter alterations in the pathogenesis of dementia (7).

The investigation of neurocognitive impairment in HIV patients is also the aim of Nguchu et al. which, exploring dynamic resting-state functional connectivity (FC), identified greater FC variability as a meaningful correlate of early cognitive impairment. Alterations in brain functional connectivity dynamics underlie cognitive impairment in different diseases (8), representing a perfect example of pathological mechanism translating across conditions. Finally, resting-state fMRI has also been applied by Li et al. to characterize the functional circuits responsible for verbal fluency deficit in major depression.

Examples of how prognostic insights might be gained *via* multi-modal imaging are offered by Andronesi et al. and Serlin et al. in two completely different settings.

Andronesi et al. investigated the prognostic value of biochemical and structural imaging biomarkers, including glutathione imaging, in patients with amyotrophic lateral sclerosis (ALS), concluding that a multiparametric panel of diverse biomarkers might help to define a brain endophenotype, useful to stratify ALS patients into more homogeneous groups for therapeutic interventions compared to clinical criteria. The integration of multiple biomarkers, optimized by the application of machine learning approaches, has recently yield promising results for patients stratification and disease staging (9, 10).

On the other hand, Serlin et al. propose optic nerve deformity as an auxiliary marker for neurological risk stratification in infants with enlarged CSF space in the context of benign external hydrocephalus. The search for easy-to-perform prognostic biomarkers is of significant importance, translating to different conditions and non-invasive methods (11, 12).

Today, as exemplified by the works reported in this Research Topic, multi-modal imaging is still based on the combination of a limited number of parameters and techniques, as data acquisition and processing require the allocation of consistent resources in terms of funds, time, and specialized personnel. In the future, thanks to technical advancements and increasing availability of data through public repositories and sharing initiatives, we will be able to integrate information from an ever-growing number of sources, expanding the boundaries of multi-modal research beyond our current perspective.

## Author Contributions

The author confirms being the sole contributor of this work and has approved it for publication.

## Conflict of Interest

The author declares that the research was conducted in the absence of any commercial or financial relationships that could be construed as a potential conflict of interest.

## Publisher's Note

All claims expressed in this article are solely those of the authors and do not necessarily represent those of their affiliated organizations, or those of the publisher, the editors and the reviewers. Any product that may be evaluated in this article, or claim that may be made by its manufacturer, is not guaranteed or endorsed by the publisher.
